# The polar-localized borate exporter BOR1 facilitates boron transport in tapetal cells to the developing pollen grains

**DOI:** 10.1093/plphys/kiaf100

**Published:** 2025-03-19

**Authors:** Keita Muro, Arisa Yamasaki, Maki Matsumoto, Yu-Ki Tanaka, Yasumitsu Ogra, Toru Fujiwara, Akira Yoshinari, Junpei Takano

**Affiliations:** Graduate School of Agriculture, Osaka Metropolitan University, Osaka 599-8531, Japan; Graduate School of Life and Environmental Sciences, Osaka Prefecture University, Osaka 599-8531, Japan; Graduate School of Life and Environmental Sciences, Osaka Prefecture University, Osaka 599-8531, Japan; Graduate School of Pharmaceutical Sciences, Chiba University, Chiba 260-8675, Japan; Graduate School of Pharmaceutical Sciences, Chiba University, Chiba 260-8675, Japan; Graduate School of Agricultural and Life Sciences, The University of Tokyo, Tokyo 113-8657, Japan; Institute of Transformative Bio-Molecules (WPI-ITbM), Nagoya University, Nagoya 464-8601, Japan; Graduate School of Agriculture, Osaka Metropolitan University, Osaka 599-8531, Japan; Graduate School of Life and Environmental Sciences, Osaka Prefecture University, Osaka 599-8531, Japan

## Abstract

Boron is an essential micronutrient required for plant cell wall integrity, as it is necessary for crosslinking the pectic polysaccharide rhamnogalacturonan II. Reproductive organs require a greater amount of boron for development and growth compared with vegetative organs. However, the mechanism by which plants distribute boron to specific organs is not fully understood. Under boron-limited conditions, the borate exporter BOR1 plays a central role in transporting boron from the roots to the shoots in Arabidopsis (*Arabidopsis thaliana*). Here, we found that *BOR1* is expressed in the tapetal cells of young anthers in unopened buds, showing polar localization toward the locule where microspores develop. Tapetum-localized BOR1 undergoes endocytosis and is subsequently degraded during anther development. BOR1 degradation occurs independently of the lysine residue at Position 590 of BOR1, which is responsible for high boron–induced ubiquitination and degradation. Loss-of-function *bor1* mutants exhibit disrupted pollen structure, causing reduced fertility under boron-sufficient conditions in the wild type. These phenotypes were rescued by supplementing with high boron concentrations. Furthermore, inflorescence stem grafting experiments suggested that BOR1-dependent boron transport in the flower is necessary for pollen development and subsequent fertilization under boron-sufficient conditions. Our findings suggest the borate exporter BOR1, together with the previously described boric acid channel NIP7;1, facilitates boron transport in tapetal cells toward the locule, thereby supporting pollen development in young anthers under boron-limited conditions.

## Teaser

A borate exporter facilitates boron transport in tapetal cells toward the locule, thereby supporting pollen development in young anthers under boron-limited conditions.

## Introduction

Boron (B) is one of the essential micronutrients for plants. The main role of B in plants is to maintain cell wall integrity by crosslinking pectin at rhamnogalacturonan II (Kobayashi et al. 1996). B deficiency causes a variety of symptoms, including root growth inhibition and reduced expansion of young leaves (Dell and Huang 1997). Moreover, B deficiency impacts crop yield and quality by affecting reproductive growth. In wheat, more B is required in reproductive growth than in vegetative growth (Rerkasem et al. 1997). B deficiency affected the development of pollen and the seed yield of low B-sensitive wheat. *Brassica napus* exhibited the typical symptom of B deficiency, “flowering without seed setting,” under B-deficient conditions (Zhang et al. 2014). Therefore, B acquisition and distribution are particularly important for reproductive growth.

Boric acid channels belonging to nodulin 26-like intrinsic proteins (NIPs) subfamily within the major intrinsic protein superfamily and borate transporters (BORs) play major roles in B transport through plant cells. In Arabidopsis roots, NIP5;1, BOR1, and BOR2 play central roles in growth under B-limited conditions (Noguchi et al. 1997; Takano et al. 2002b, 2006; Miwa et al. 2013). NIP5;1 is polarly localized to the outer (soil side) plasma membrane (PM) domain of Arabidopsis root epidermal cells, while BOR1 and BOR2 are localized to the inner (stele side) PM domain of root epidermal and endodermal cells (Takano et al. 2010; Miwa et al. 2013). The polar localization is important for efficient directional transport of B into the stele (Yoshinari et al. 2019). In addition to transporting B in roots, BOR1 was reported to play a role in the preferential distribution of B into young leaves and reproductive organs (Noguchi et al. 1997; Takano et al. 2001). BOR1 is expressed and localized to the PM in a polar manner in various above-ground tissues, including epidermis of cotyledon and endodermis of hypocotyl (Yoshinari et al. 2016). This suggests that B is directionally transported in different cells. Other members of the NIP subfamily have also been reported to facilitate B transport in plants. NIP6;1 is expressed in the phloem tissue of stem nodes and functions in preferential B transport to growing tissues (Tanaka et al. 2008). NIP7;1 is mainly expressed in tapetal cells in anthers and plays roles in pollen development and fertility under low B conditions (Routray et al. 2018).

*BOR1* gene was originally identified from the Arabidopsis mutant with reduced fertility under a standard growth condition for Arabidopsis (30 *μ*m; Noguchi et al. 1997). The fertility of this mutant, *bor1-1*, was restored by adding 150 *μ*m of B. Reduced fertility in *bor1-1* mutant was shown to be due to female sterility, based on the results of reciprocal crosses. Under a standard B condition (30 *μ*m), wild-type pollen failed to fertilize the pistil of *bor1-1*, whereas *bor1-1* pollen could fertilize emasculated wild-type plants. It is presumably due to low concentrations of B in female reproductive organs caused by the defect in root-to-shoot translocation and preferential distribution of B in the *bor1-1* mutant (Noguchi et al. 1997; Takano et al. 2001). However, the precise role of BOR1 in reproductive growth remains unclear.

In this study, we examined the expression and localization of BOR1 in flowers by analyzing transgenic plants expressing *BOR1-GUS* and *BOR1-GFP*. BOR1 was expressed predominantly in the tapetum cells in young anthers and localized to the locule side of the PM. Next, we analyzed the phenotypes of *bor1* mutants. When grown under a standard B condition (20 *μ*m), *bor1* plants displayed decreased fertility, as reported previously (Noguchi et al. 1997). Pollen grains in these plants were morphologically defective, displaying disordered pollen walls and collapsed pollen shapes. By inflorescence stem grafting, the defective pollen morphology or sterility was not observed in the Col-0 scions grafted onto *bor1-1* rootstocks. Our study suggests that tapetum BOR1 plays a role in supplying B to developing pollen in the locule.

## Results

### BOR1 is expressed in young anthers under low to normal B conditions

BOR1 is expressed in various tissues and shows polar localization in the PM (Yoshinari et al. 2016). The *bor1-1* mutant shows reduced vegetative growth under 0.3 and 3 *μ*m B conditions but shows comparable growth with Col-0 under standard or higher B conditions (12, 30 *μ*M, or more; Noguchi et al. 1997). By contrast, the reproductive growth of *bor1-1* is impaired under a standard B condition (30 *μ*m), showing normal inflorescence but poor silique development, which is restored under a higher B condition (150 *μ*m). This study is in line with the studies in other crop plants that revealed the specific importance of B in reproductive growth (Rerkasem et al. 1997; Zhang et al. 2014). These studies motivated us to investigate the role of BOR1 in reproductive growth.

**Figure 1.**
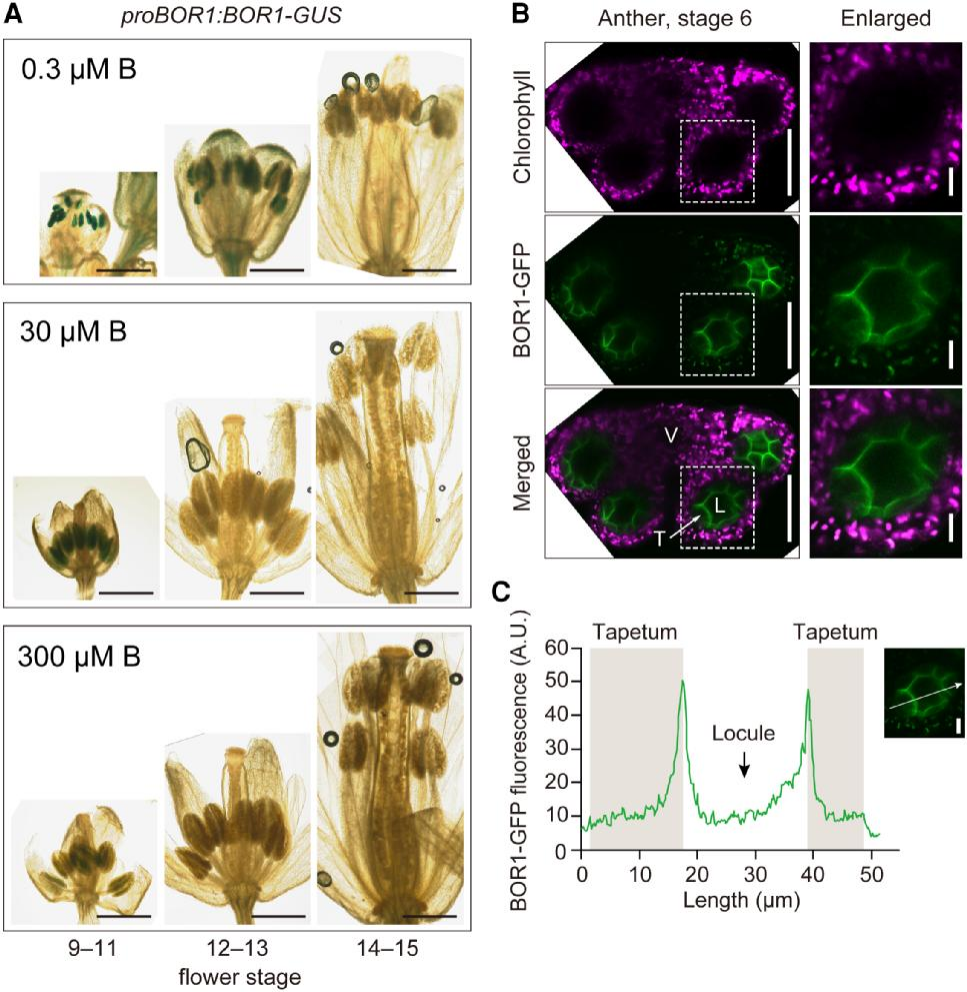
BOR1 is expressed in tapetum cells and localized in the locule-facing domain of the PM. **A)** GUS staining of flowers at various developmental stages in *proBOR1:BOR1-GUS* plants grown with hydroponic medium containing 0.3, 30, or 300 *µ*m boric acid. Flower development stages are indicated below (Smyth et al. 1990; Bowman et al. 1991). Scale bars indicate 500 *µ*m. **B)** Confocal image of BOR1-GFP and autofluorescence derived from chlorophyll in the anther of *proBOR1:BOR1-GFP* plants at Stage 6. The plants were grown on rockwool supplied with 0.3 *µ*m boric acid. Scale bars indicate 50 *µ*m (left) and 10 *µ*m (right, enlarged), respectively. **C)** Quantification of fluorescence intensity along a line indicated in the micrograph on the right, which is the same as the one shown in **B)**. BOR1-GFP showed the highest fluorescence at the locule-facing domain of the PM. AU, arbitrary units; V, vascular region; L, locule; T, tapetum.

To elucidate the role of BOR1 in reproductive growth, we examined the BOR1 protein accumulation in the inflorescence through GUS histochemical staining in plants expressing *BOR1-GUS* translational fusion under the control of the *BOR1* promoter (Miwa et al. 2013). In the shoots of the transgenic plants grown under a low B condition (3 *μ*m), GUS histochemical staining was observed only in the flowers and young part of the stems (Supplementary Fig. S1). We then examined the *BOR1-GUS* staining in the flowers more precisely, under low to high B conditions (Fig. 1A). GUS staining was observed in the anthers in young buds when grown under 0.3 to 30 *μ*m B conditions. On the other hand, GUS staining was faintly observed in the anthers in the inflorescences of the plants grown under 300 *μ*m B condition. These results suggested that BOR1 is accumulated in young anthers in unopened buds, under low to standard B conditions.

### BOR1 shows polar localization in tapetum cells

To examine the localization of BOR1 at a cellular or subcellular level, we conducted confocal microscopy of anther tissues using pro*BOR1*:*BOR1-GFP* expressing plants (Takano et al. 2010). Under a low B condition (0.3 *μ*m), fluorescence of BOR1-GFP was specifically observed in the tapetum cells, the single cell layer surrounding the locule (Fig. 1B). Intriguingly, BOR1-GFP was polarly localized in the PM, biased toward the locule side, in anther Stages 5 to 7 (Fig. 1, B and C). The polar localization of BOR1 toward the locule-facing domain in tapetal cells suggests directional transport of B into the locule.

**Figure 2.**
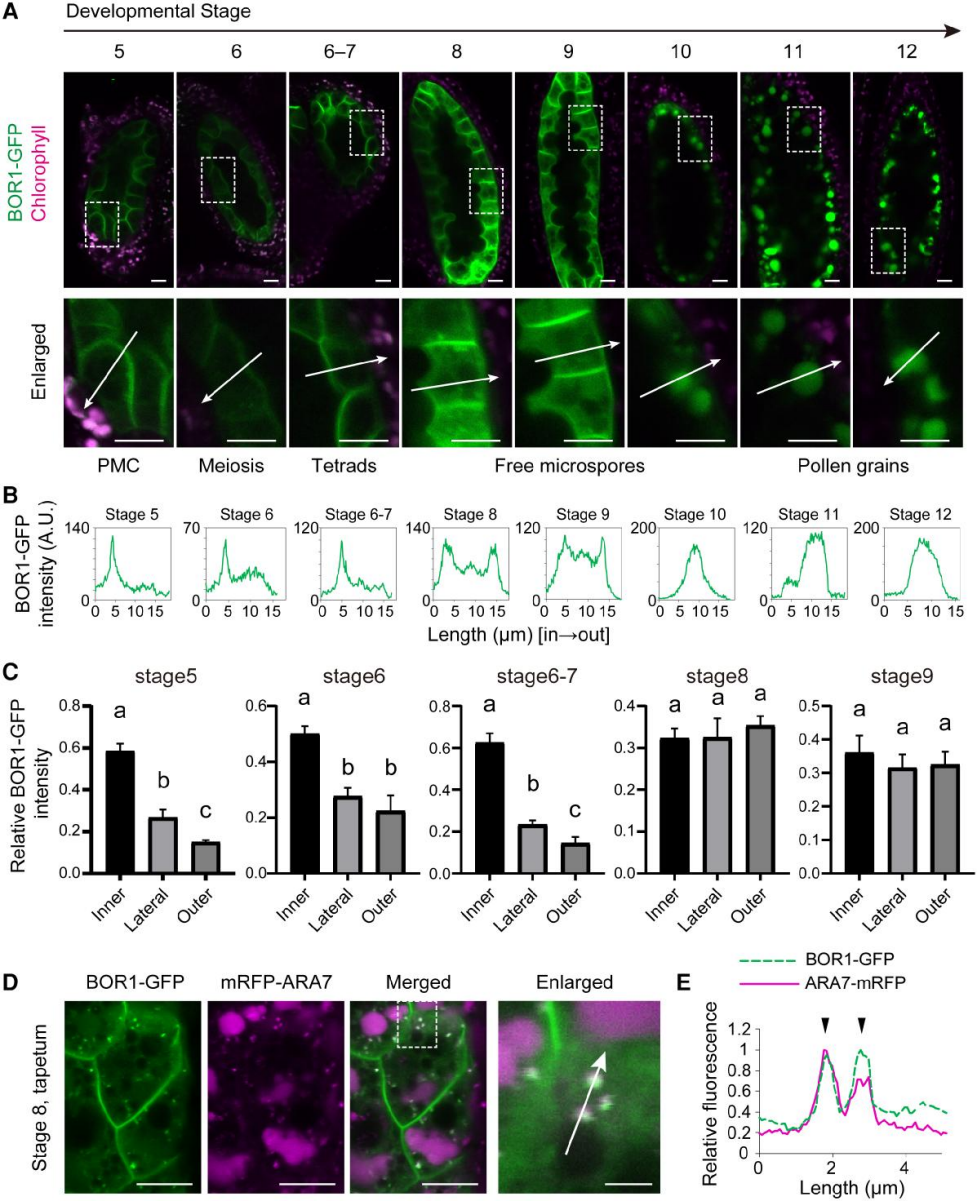
BOR1 is transported into the vacuole in tapetum cells in the later developmental stages. **A)** Confocal images of BOR1-GFP in tapetal cells and chlorophyll fluorescence in outer cells in anthers at various developmental stages. Plants were grown hydroponically with the medium containing 30 *μ*m B for 4 wk and then shifted to 3 *μ*m B medium. The bottom panels show enlarged images of the area indicated in the images above. Pollen developmental events corresponding to each anther stage are indicated below. Scale bars = 10 *µ*m. **B)** Plots of fluorescence intensity along the lines indicated in the enlarged images in **A)**. The plots represent the fluorescence intensity along the lines from the inner (facing the locule) to the outer (facing the middle layer) sides of the tapetum cells. **C)** Relative BOR1-GFP signal on inner, lateral, and outer sides of the tapetal PM at different anther stages. Intensities were normalized by dividing by the sum of inner, lateral, and outer signals. Values are means ± Sd (*n* = 3 cells each). Different letters indicate significant differences (*P* < 0.05) by Tukey's multiple comparisons test. **D)** Confocal images of tapetum in Stage 8 expressing BOR1-GFP and mRFP-ARA7. Scale bars represent 10 and 2 *µ*m (enlarged). **E)** Plots of relative fluorescence intensities of BOR1-GFP and mRFP-ARA7 along the line indicated in **D)**. Arrowheads indicate peaks of intensity that coincide with each other. PMC, pollen mother cell; AU, arbitrary units.

To investigate the temporal role of BOR1 during anther development, we captured snapshots of BOR1-GFP fluorescence at various developmental stages of the anther (Fig. 2A) and quantified signals along lines across tapetal cells (Fig. 2B) and in the inner, lateral, and outer PM domains (Fig. 2C) to assess subcellular and polar localization. Anther development stages were judged based on the morphology of microspores, pollen, and tapetal cells (Sanders et al. 1999). At Stages 5 to 7, when microspore mother cells divide into tetrads, BOR1-GFP was localized to the PM in a polar manner toward the locule. At Stages 8 to 9, when microspores are released from tetrads and mature, the polar localization was less pronounced, and the presence of BOR1-GFP in intracellular compartments was increased. It should be noted that tapetal cells are apparently intact at Stage 8 (Supplementary Fig. S2). Later, at Stages 10 to 12, when pollen undergo mitosis, BOR1-GFP was nearly undetectable in the PM, and the GFP fluorescence was observed in the vacuolar lumen. Collectively, BOR1 is likely functional in the locule-facing region of the tapetal PM in young anthers at Stages 5 to 7 for directional B transport to the locule and subsequently internalized and degraded in vacuoles during later anther stages before the degradation of tapetum cells.

The evidence that BOR1 is expressed in tapetal cells prompted us to examine the behavior of the tapetum-specific boric acid channel NIP7;1 during anther development. We assessed the subcellular localization of NIP7;1-mGFP in developing anthers under a low B (3 *µ*m) condition (Supplementary Fig. S3). At Stages 5 to 7, the localization of NIP7;1-mGFP was ambiguous. At Stages 8 to 9, NIP7;1-mGFP was observed on the PM, as previously reported (Routray et al. 2018). At later stages from 10, the NIP7;1-mGFP fluorescence was predominantly observed in the vacuolar lumen, similar to the situation with BOR1. Collectively, BOR1 and NIP7;1 likely function in the tapetal cells during the early stages of anthers and degrade in the later stages.

### BOR1 is degraded via endocytic degradation pathway

To clarify how BOR1 is sorted to the vacuole during anther development, we examined the colocalization of BOR1-GFP and an endosome marker mRFP-ARA7 (Ueda et al. 2004; Inada et al. 2016) in transgenic plants coexpressing these fluorescent-tagged proteins. In the anthers at Stage 8, BOR1-GFP and mRFP-ARA7 showed colocalization at the punctate structures (Fig. 2, D and E; Supplementary Fig. S4). Therefore, BOR1 is likely transported to the vacuole through the endocytic pathway in the later stage of tapetal cells.

**Figure 3.**
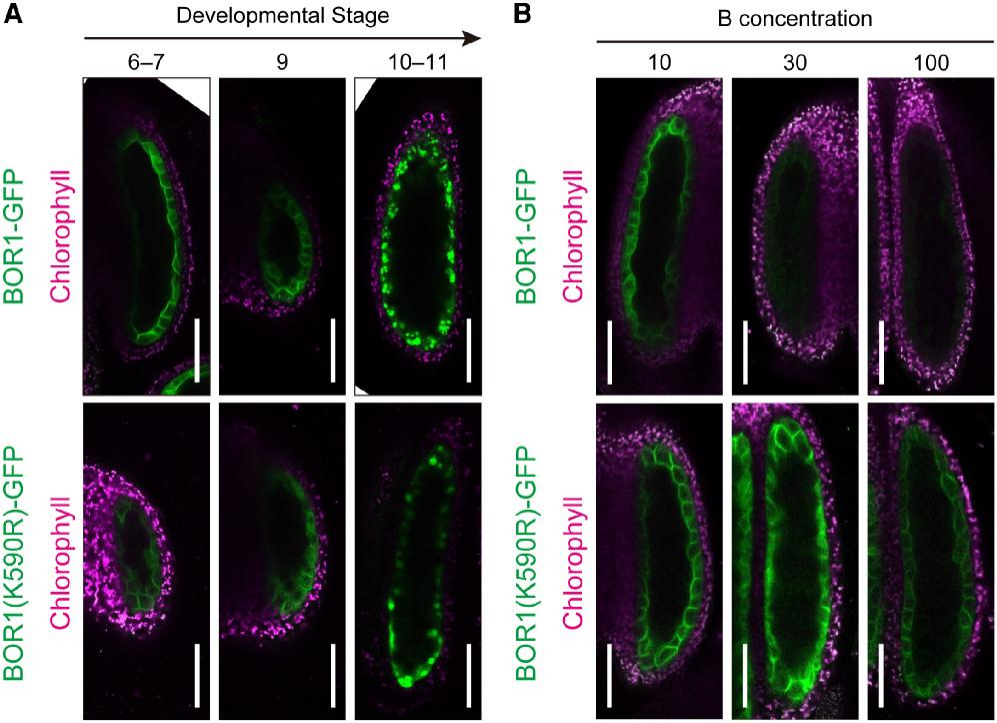
Stage- and B-dependent degradation of BOR1. **A)** Subcellular localization of BOR1-GFP (top) or BOR1(K590R)-GFP (bottom) in anthers of Stages 6, 7, 9, 10, and 11. Plants were grown hydroponically with the medium containing 3 *μ*m B. Scale bars represent 50 *μ*m. **B)** GFP fluorescence in the anthers of the plants expressing BOR1-GFP or BOR1(K590R)-GFP at Stages 6 to 7. Plants were grown hydroponically with the medium containing 10, 30, or 100 *μ*m B. Scale bars represent 50 *μ*m. Representative images from more than 3 plants are shown.

In root cells, BOR1 is transported into the vacuole for degradation in response to high concentrations of B via the endocytic pathway, which is dependent on the ubiquitination of the lysine 590 residue (K590) of BOR1 (Kasai et al. 2011; Yoshinari et al. 2021). To examine whether the same mechanism is involved in the degradation of BOR1 during anther development, we analyzed the behavior of a BOR1-GFP variant with a lysine to arginine substitution. During the anther development, BOR1(K590R)-GFP was internalized and degraded in a similar manner to wild-type BOR1-GFP (Fig. 3A; Supplementary Fig. S2). Therefore, the vacuolar sorting of BOR1 in the later stage of anther development is independent of the ubiquitination of K590.

To investigate whether the ubiquitination of K590 is required for the B-dependent degradation of BOR1 in the anther, we compared the abundance of wild-type BOR1-GFP and BOR1(K590R)-GFP under different B conditions (Fig. 3B). Under a 10-*μ*m B condition, both wild-type BOR1-GFP and BOR1(K590R)-GFP were observed in the PM of tapetal cells. On the other hand, wild-type BOR1-GFP fluorescence was dramatically reduced under 30 and 100 *μ*m B conditions, while the fluorescence of BOR1(K590R)-GFP was consistently detected under the same conditions. These results suggest that BOR1 in tapetum cells is degraded under higher B conditions through the ubiquitin-dependent endocytic pathway as previously shown in the root cells.

### *Bor1* mutants show reduced fertility and irregular pollen morphology

**Figure 4.**
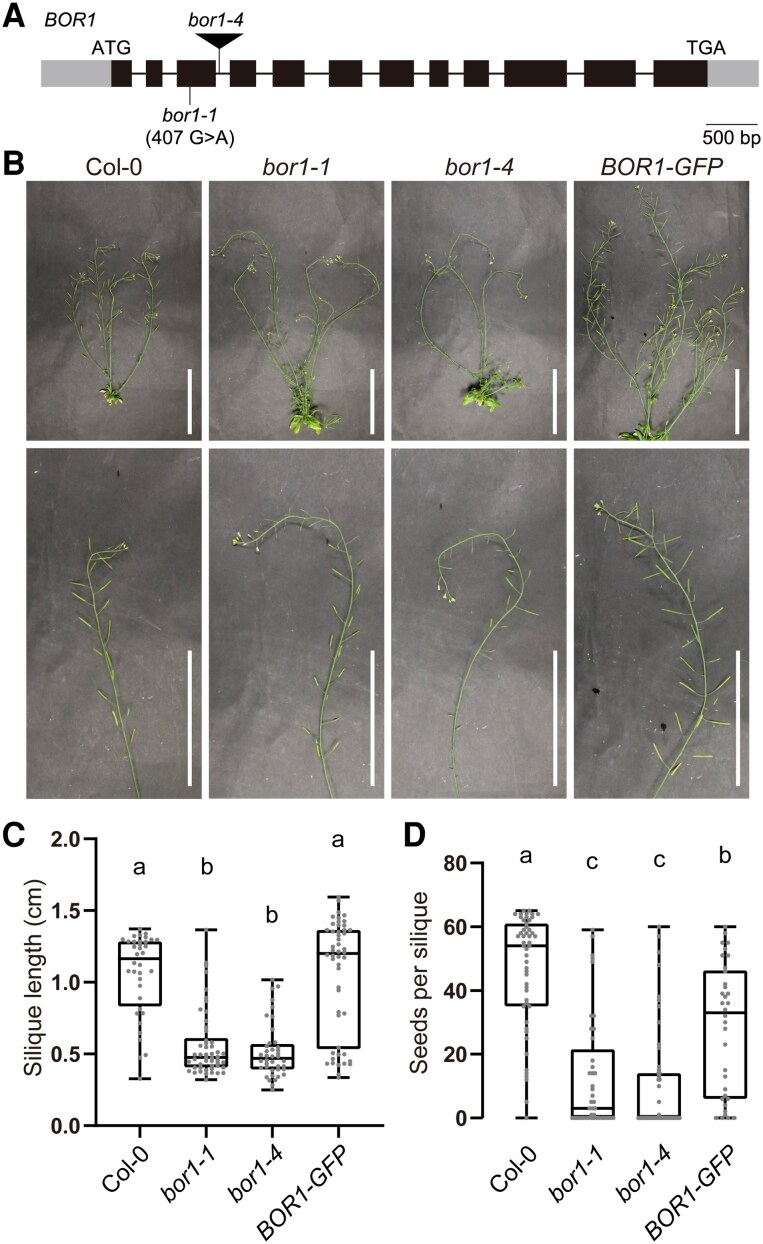
*Bor1* mutants show reduced fertility. **A)** Schematic illustration of the *BOR1* gene showing the point mutation and T-DNA insertion sites of the *bor1* mutants. Exons, introns, and UTRs are indicated by black boxes, black bars, and gray boxes, respectively. **B)** Representative pictures of the inflorescence stems of Col-0, *bor1* mutants, and transgenic *bor1-1* plants expressing *BOR1-GFP*. Plants were grown hydroponically with the medium containing 100 *μ*m B until bolting and then shifted to 20 *μ*m B condition. Scale bars represent 10 cm. **C)** Silique lengths of Col-0, *bor1* mutants, and transgenic *bor1-1* plants expressing *BOR1-GFP* grown as indicated in **B)**. *n* = 36 to 50 siliques from 4 to 5 plants. Different letters indicate significant differences (*P* < 0.0001) by Tukey's multiple comparisons test. **D)** Seed number per silique of Col-0, *bor1* mutants, and transgenic *bor1-1* plants expressing BOR1-GFP grown as indicated in **B)**. *n* = 41 to 52 siliques from 3 plants. Different letters indicate significant differences (*P* < 0.01) by Tukey's multiple comparisons test. In the box plots, center line, median; box limits, lower and upper quartiles; dots, individual data points; whiskers, highest and lowest data point.

*Bor1-1* mutant was reported to exhibit female sterility under a 30-*μ*m B condition, which is a standard condition for Arabidopsis growth in laboratories (Noguchi et al. 1997). Nevertheless, the above-mentioned expression and localization pattern of BOR1 in anther suggested the involvement of BOR1 in the male reproductive organ. To elucidate the role of BOR1 in reproductive tissues, we examined the phenotypes of *bor1* mutant inflorescences in detail using *bor1-1* and a T-DNA insertion allele *bor1-4* (SALK_022077, Fig. 4A). Plants were grown hydroponically with liquid medium containing 100 *μ*m B until bolting and then transferred to standard B (20 *μ*m). In this condition, both *bor1-1* and *bor1-4* developed shorter siliques containing fewer seeds, compared with wild-type Col-0 plants, indicating reduced fertility (Fig. 4, B to D). These phenotypic defects were rescued at least partially in the transgenic *bor1-1* plants harboring *proBOR1*:*BOR1-GFP*. Additionally, we observed the morphology of tapetal cells at Stages 5 to 7 from Col-0 and *bor1-1* grown under a standard B (20 *μ*m) condition after bolting (Supplementary Fig. S5). The tapetal cells in the *bor1-1* mutant showed no obvious morphological defect compared with Col-0, suggesting that BOR1 is not involved in the development or maintenance of tapetal cells.

**Figure 5.**
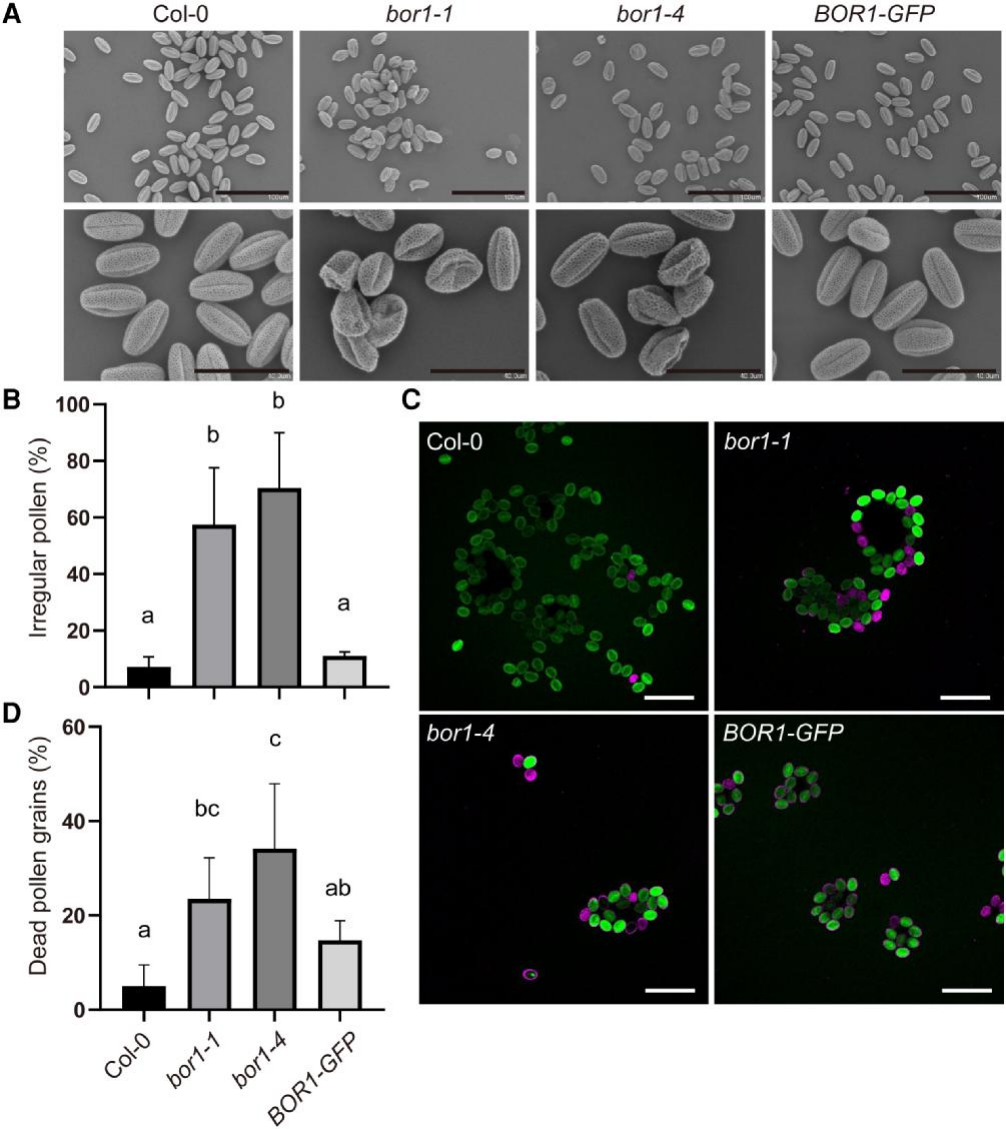
*Bor1* mutants produce defective pollen grains. **A** to **D)** Pollen phenotypes of Col-0, *bor1-1*, *bor1-4*, and transgenic *bor1-1* plants expressing *BOR1-GFP*. Plants were grown hydroponically with the medium containing 100 *μ*m B until bolting and then shifted to 20 *μ*m B **A** and **B)** or 30 *μ*m B **C** and **D)** medium. **A)** Representative scanning electron microscope images of the pollen grains. The images in the top and bottom rows are independent of each other. Bars represent 100 (top) and 40 *μ*m (bottom). **B)** Percentage of the pollen grains with irregular morphology. Values are means ± Sd (*n* = 3 plants with 64 to 291 pollen grains each). Different letters indicate significant differences (*P* < 0.05) by Tukey's multiple comparisons test. **C)** Representative images of pollen grains stained by PI (magenta, indicating dead pollen) and FDA (green, indicating alive pollen). Bars represent 100 *μ*m. **D)** Percentage of dead pollen grains assessed through PI/FDA staining. Values are means ± Sd (*n* = 5 plants with 81 to 301 pollen grains each). Different letters indicate significant differences (*P* < 0.05) by Tukey's multiple comparisons test.

To clarify the role of BOR1 in anthers, we then examined the phenotypes of pollen grains in the *bor1* mutants. Scanning electron microscopy (SEM) revealed that a major portion of pollen grains from the *bor1* mutant plants supplied with standard B (20 *μ*m) after bolting exhibited irregular pollen wall patterns and collapsed structures (Fig. 5, A and B). On the other hand, pollen grains from *bor1* plants grown under a 100-*μ*m B condition showed normal morphology (Supplementary Fig. S6). These results indicate that BOR1 is required for pollen development under standard B conditions.

To test pollen viability, we stained the pollen grains using propidium iodide (PI) and fluorescein diacetate (FDA), using the plants grown under a normal B condition (30 *μ*m) after bolting (Fig. 5, C and D). Dead pollen grains stained by PI but not by FDA were more frequently observed in *bor1-1* and *bor1-4* plants (24% and 34%) than in Col-0 (5%). The percentage of dead pollen grains in the transgenic *bor1-1* plants harboring *proBOR1*:*BOR1-GFP* (15%) was lower than in *bor1-1*, although the difference was not statistically significant.

**Figure 6.**
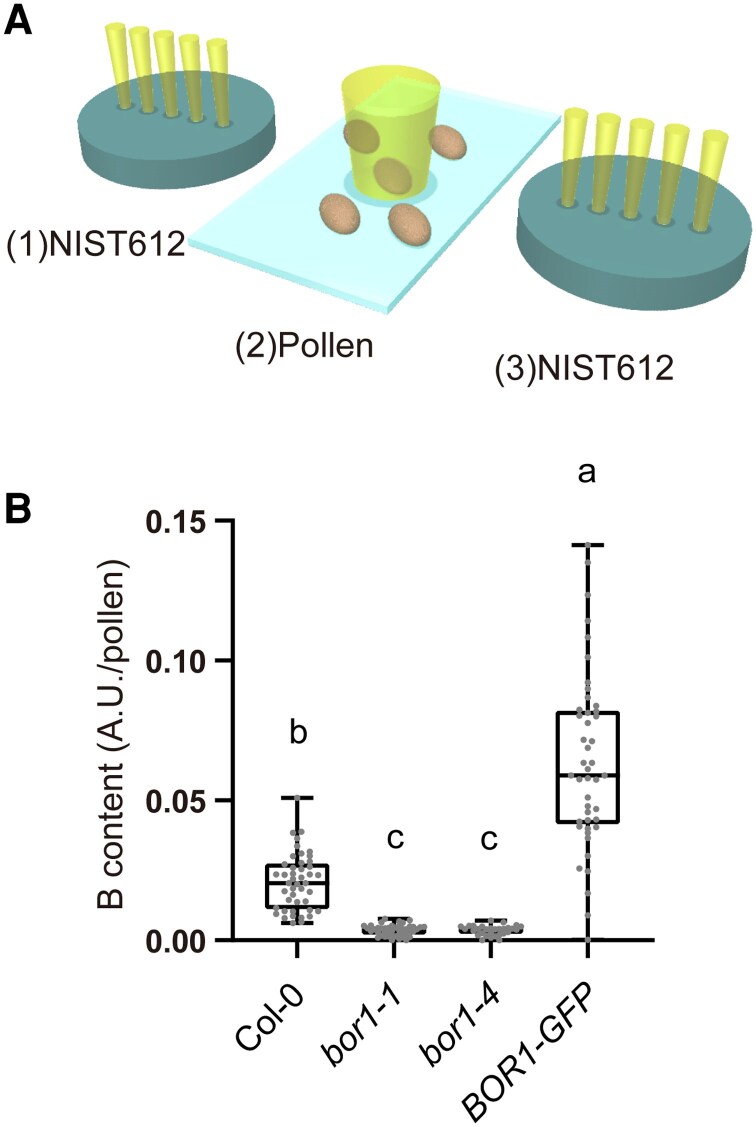
LA-ICP-MS analysis of B in pollen grains. **A)** Schematic illustration of the analysis of B contents of pollen grains by LA-ICP-MS. Pollen grains were individually ablated using a pulsed laser, and the resulting sample aerosols were analyzed by ICP-MS. Changes in instrumental sensitivity were corrected using the signal intensity of B obtained from the NIST 612 glass standard. **B)** B content in a pollen analyzed by LA-ICP-MS. Samples were taken from Col-0, *bor1-1*, *bor1-4,* and transgenic *bor1-1* plants expressing *BOR1-GFP*. Plants were grown hydroponically with the medium containing 100 *μ*m B until bolting and then shifted to 20 *μ*m B medium. *n* = 30 to 45 pollen from 2 to 3 plants. Different letters indicate significant differences (*P* < 0.001) by Tukey's multiple comparisons test. In the box plots, center line, median; box limits, lower and upper quartiles; dots, individual data points; whiskers, highest and lowest data point. AU, arbitrary units.

We hypothesized that reduced B content in microspores in *bor1* mutants resulted in defective pollen development. To evaluate the abundance of B in individual pollen grains, we established a technique to measure the B content in the pollen grains employing laser ablation inductively coupled plasma MS (LA-ICP-MS, Fig. 6A). Pollen grains from *bor1* mutants supplied with standard B (20 *μ*m) after bolting contained significantly less B than those from Col-0 (83% less than in Col-0, Fig. 6B). On the other hand, the B content in the pollen from the *bor1-1* complemented line with *proBOR1:BOR1-GFP* was even higher than in those of Col-0. Collectively, these results suggested that BOR1 is required for pollen development and fertility, by supplying B to developing pollen under low to normal B conditions.

### *BOR1* expression in inflorescence rescues the pollen morphology

**Figure 7.**
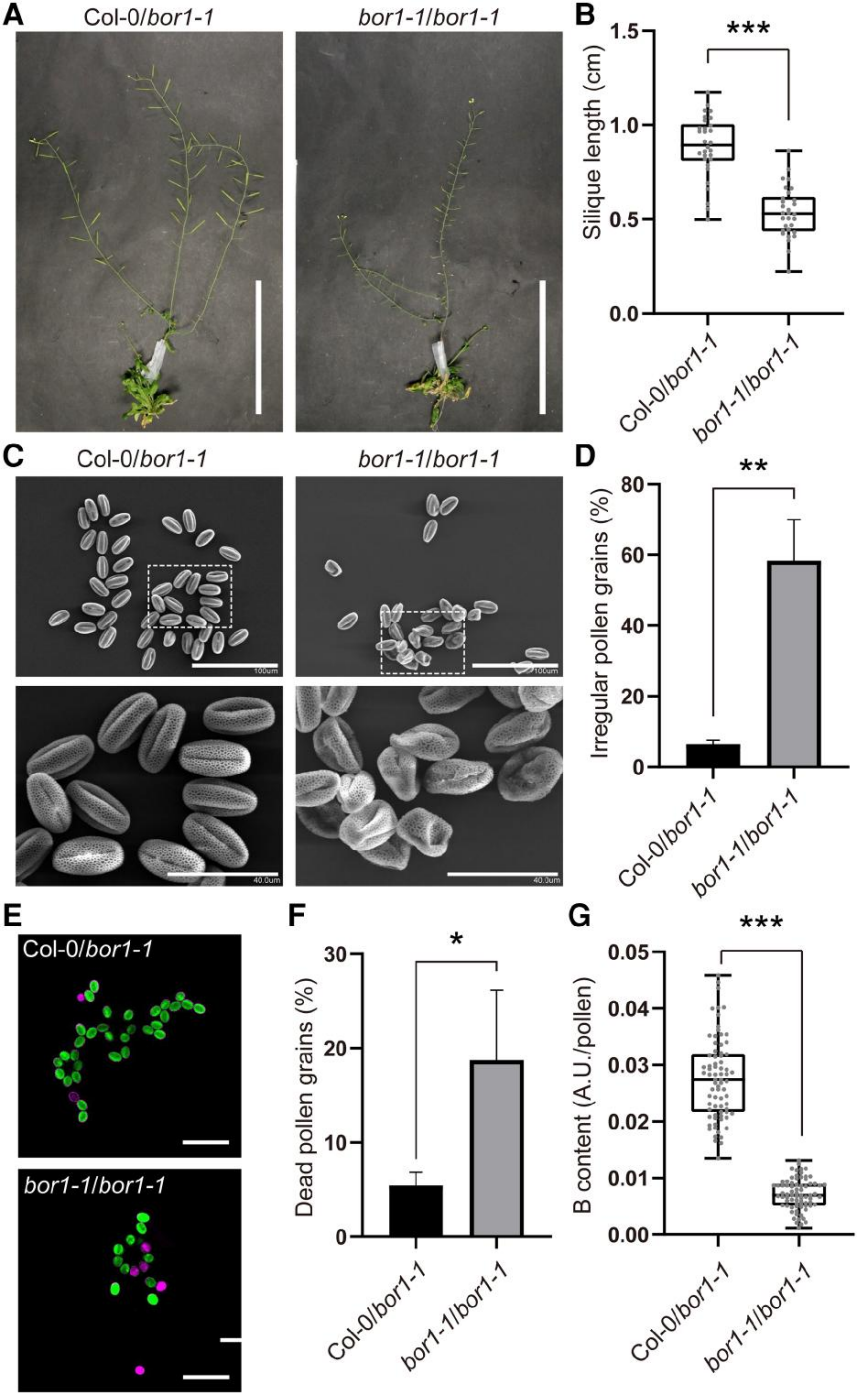
BOR1 in the inflorescence stem rescued the defective fertility and pollen of the *bor1-1* mutant. **A** to **G)** Phenotypes of the plants grafted at the inflorescence stems. Col-0 or *bor1-1* scions were grafted onto *bor1-1* rootstocks (indicated as Col-0/*bor1-1* and *bor1-1*/*bor1-1*, respectively). Plants were grown hydroponically with the medium containing 100 *μ*m B until grafting and then shifted to 20 *μ*m B condition. **A)** Representative pictures of the inflorescence stems of the grafted plants. Scale bars represent 10 cm. **B)** Silique lengths of the grafted plants. Data are means ± Sd (*n* = 30 siliques from 3 plants). Asterisk represents a significant difference according to Student's *t*-test (***P < 0.001). **C)** Representative scanning electron microscope image of the pollen grains from the grafted plants. Enlarged images of the area shown in the top panels are displayed in the bottom panels. Scale bars represent 100 (top) and 40 *μ*m (bottom). **D)** Percentage of the pollen grains with irregular morphology. Data are means ± Sd (*n* = 3 plants with 101 to 212 pollen grains each). Asterisk represents a significant difference according to Student's *t*-test (**P < 0.01). **E)** Representative images of pollen grains from the grafted plants. Pollen grains were stained by PI (magenta, which stains dead pollen) and FDA (green, which stains alive pollen). Bars represent 100 *μ*m. **F)** Percentage of dead pollen grains assessed through PI/FDA staining. Values are means ± Sd (*n* = 3 plants with 38 to 237 pollen grains each). Asterisk represents a significant difference according to Student's *t*-test (*P < 0.05). **G)** B content in a pollen analyzed by LA-ICP-MS. Data are means ± Sd (*n* = 75 pollen grains from 5 plants). Asterisk represents significant difference according to Student's *t*-test (***P < 0.001). In the box plots, center line, median; box limits, lower and upper quartiles; dots, individual data points; whiskers, highest and lowest data point. AU, arbitrary units.

In a previous inflorescence stem grafting experiment, Col-0 scions on *bor1-1* rootstocks rescued the seed set under a normal B condition (Takano et al. 2002a). This result suggested the important function of BOR1 in the reproductive tissues in addition to the function in xylem loading in roots (Noguchi et al. 1997). Here, we reproduced the results and analyzed the phenotype in more detail. When *bor1-1* rootstocks and *bor1-1* scions were grafted, the plants developed shorter siliques similar to nongrafted *bor1-1* plants, under a standard B condition (20 *μ*m, Fig. 7, A and B). On the other hand, Col-0 scions on *bor1-1* rootstocks developed longer siliques under the same condition as reported previously. We then analyzed morphology, viability, and B content of pollen grains from these grafted plants. SEM demonstrated that most of the pollen grains from Col-0 scions grafted onto *bor1-1* rootstocks exhibited regular morphology similar to nongrafted Col-0 (Fig. 7, C and D). By contrast, many pollen grains from *bor1-1* scions grafted on *bor1-1* rootstocks exhibited aberrant morphology similar to those of nongrafted *bor1-1*. PI/FDA staining revealed that the pollen from the grafted plants with Col-0 scion had higher viability (5% dead) compared with that from *bor1-1* scion (19% dead, Fig. 7, E and F). These results indicate that B transport by BOR1 in the inflorescence is necessary for pollen development. Moreover, LA-ICP-MS analysis demonstrated that pollen grains from the Col-0 scion contained significantly higher B than the *bor1-1* scion (Fig. 7G). Taken together, BOR1 in the inflorescence plays a vital role in transporting B to the locule to support pollen development under low to standard B conditions.

## Discussion

BOR1 plays an important role in root-to-shoot translocation of B under low B conditions, through directional transport of B toward the root stele. Additionally, BOR1 is expressed in cotyledon and hypocotyl (Yoshinari et al. 2016). In the current study, we demonstrated the expression of BOR1 in tapetal cells in anthers. Tapetal cells form a layer surrounding the locule of the anther and play pivotal roles in supplying various materials required for microspore/pollen development (Pacini et al. 1985). Several transporters have been reported to function in the tapetum. For example, an ATP-binding cassette transporter ABCG26 transports sporopollenin precursors required for exine formation (Quilichini et al. 2010; Choi et al. 2011; Dou et al. 2011; Kuromori et al. 2011; Quilichini et al. 2014). A magnesium transporter MGT5 functions in the tapetal PM and exports magnesium into the locule (Xu et al. 2015). NIP7;1 is also required for pollen development under low B conditions (Routray et al. 2018). Our results suggest the role of a borate transporter BOR1 in supplying B from the tapetum to the locule.

Tapetal cells are formed by periclinal division from inner secondary parietal cells in young anthers (Sanders et al. 1999). After supplying various materials into the locule through transporters and by exocytosis, tapetal cells degenerate by programmed cell death (PCD) and release the cell components. In our observations, BOR1 in the tapetum was internalized and transported into vacuole prior to PCD (Fig. 2A; Supplementary Fig. S4). Interestingly, this internalization of BOR1 during development was independent on lysine 590 in BOR1, which is ubiquitinated and necessary for high B–induced vacuolar sorting (Fig. 3A; Supplementary Fig. S4; Kasai et al. 2011; Yoshinari et al. 2021). On the other hand, the regulation of BOR1 protein level in response to B concentration depended on the K590 residue in tapetal cells, similar to the case in root cells (Fig. 3B). These results highlight that the development-dependent internalization of BOR1 is distinct from the B-dependent internalization process, which functions to prevent excessive B transport, and that both processes operate in tapetal cells. Development-dependent degradation may play an important role in supplying amino acids to microspores.

The mechanism underlying the development-dependent internalization of BOR1 remains unknown. Degradation of PM-localized proteins may depend on autophagy. In the root cap, where cells are continuously replaced, autophagy is activated prior to developmentally regulated PCD (Feng et al. 2022). However, in Arabidopsis, mutants of the autophagy-related *ATG* genes exhibit normal reproductive growth and fertility under standard growth conditions, suggesting that autophagy is dispensable for sexual reproduction (Hanaoka et al. 2002; Norizuki et al. 2020; Zhao et al. 2020). As BOR1 was localized in the endosomes labeled with mRFP-ARA7 (Fig. 2, A, D, and E; Supplementary Fig. S4), its internalization and vacuolar degradation are likely controlled by endocytic trafficking. Unlike BOR1, ABCG9 and ABCG16 show no obvious change in subcellular localization between Stages 6 and 8 in tapetal cells (Liu et al. 2022). Therefore, it is possible that BOR1 is selectively internalized during anther development via the endocytic pathway, rather than as part of a general internalization process for membrane proteins. As an example of endocytosis and vacuolar degradation related to tissue development, the degradation of an auxin transporter PIN1 during lateral root emergence was reported (Marhavý et al. 2011). This internalization is induced by cytokinin and is affected by the phosphorylation status of PIN1 (Marhavý et al. 2011; Marhavý et al. 2014). It is tempting to study the mechanism underlying the internalization of transport proteins in the tapetum in the future.

BOR1 is localized to the inner side of the tapetal PM during anther Stages 5 to 7. During these stages, microspore mother cells undergo meiosis and the primexine layer of the pollen wall begins to develop (Sanders et al. 1999; Ariizumi and Toriyama 2011). Similarly, NIP7;1 shows high expression during anther Stages 5 to 9 (Routray et al. 2018). Thus, B transport in tapetum is active during early pollen development. During anther development, tapetal cells are isolated from the outer cell layers both symplastically and apoplastically from the onset of meiosis (Stage 6; Truskina et al. 2022). Therefore, it is unlikely that NIP7;1 facilitates B transport into tapetal cells from outer cell layers after Stage 6. Among the layers or structures in the pollen wall, the intine layer is thought to require B, as it is primarily made of pectin, along with cellulose and structural proteins. In Arabidopsis, deposition of the intine layer begins after tetrads degenerate into individual microspores (anther Stage 8; Sanders et al. 1999; Ariizumi and Toriyama 2011). In the B-deficient wheat, pollen morphology was normal before the stage when intine deposition occurs (Dell and Huang 1997; Rerkasem et al. 1997). Some mutants with disordered intine formation are reported to show severe defects in fertility (Vizcay-Barrena and Wilson 2006; Mi et al. 2022). Taking these factors into account, we propose the following hypothetical model of B transport during pollen development: B reaches the anthers and enters the tapetal cells until Stage 6, at which point the tapetal cells are isolated from the outer cell layers. During Stages 5 to 7, B in tapetum is exported toward the locule by polarly localized BOR and is used in the deposition of the pollen intine layer starting from stage 8. Although subcellular localization of NIP7;1 was unclear, it may function in B transport into tapetal cells at very early stages, or facilitating the transport of B between tapetal cells. After Stage 8, BOR1 undergoes endocytosis and degradation and active B supply toward the locule ceases.

We demonstrated that grafting the inflorescence stem of the Col-0 onto the *bor1-1* rootstock restored the B content and structure of pollen grains, as well as fertility (Fig. 7). Considering the major expression pattern of BOR1 in tapetal cells in the reproductive organs (Fig. 1; Supplementary Fig. S1), it is suggested that BOR1 in the tapetum plays a major role in transporting B for pollen development. It should be noted that the *bor1-1* mutant was previously determined to show female sterility through reciprocal crosses between the Col-0 wild-type and the *bor1-1* mutant grown under a standard B condition (30 *µ*m; Noguchi et al. 1997). The successful fertilization using *bor1-1* pollen grains was probably due to the presence of both normal and defective pollen grains in the *bor1-1* mutant (Fig. 5).

The acquisition and distribution of B are particularly important for reproductive growth and yield in crop plants (Jothi and Takano 2023). Maize *ROTTEN EAR* (*BOR1* homolog), *TLS1* (*NIP5;1* homolog), and rice *NIP3;1* are expressed in various organs, including reproductive tissues, and mutations in these genes have been shown to affect reproductive growth (Chatterjee et al. 2014; Durbak et al. 2014; Chatterjee et al. 2017; Zhou et al. 2021). In *B. napus*, expression of *BOR1* homologs and *NIP5;1* homologs is upregulated in reproductive tissues under B limitation, highlighting the importance of B transport in these tissues (Verwaaijen et al. 2023). Our results, along with previous reports on NIP7;1, suggest the important roles of a borate transporter and boric acid channel in tapetal cells supplying B toward pollen under low to standard B conditions. Our research provides important knowledge for future breeding to ensure a stable food supply in regions with a deficiency of B.

## Materials and methods

### Plant materials

The *Arabidopsis bor1-1* mutant was originally obtained by ethyl-methane sulfonate mutagenesis and isolated by backcrossing to Col-0 in previous studies (Ishikawa et al. 1991; Noguchi et al. 1997). T-DNA insertion mutant *bor1-4* (SALK_022077) was obtained from the Arabidopsis Biological Resource Center. *proBOR1:BOR1-GUS*/*bor1-1* (Miwa et al. 2013)*, proBOR1:BOR1-GFP*/*bor1-1* (Takano et al. 2010)*, proBOR1:BOR1(K590R)-GFP*/*bor1-1* (Yoshinari et al. 2021), and *proNIP7;1:NIP7:1-mGFP* (Routray et al. 2018) transgenic plants were previously reported. To generate plants expressing both BOR1-GFP and mRFP-ARA7, *proBOR1:BOR1-GFP*/*bor1-1* (Takano et al. 2010) and mRFP-ARA7 (Inada et al. 2016) plants were crossed. The plants in F2 generation were used for the observation.

### Plant growth analysis

The composition of the plant growth medium except for boric acid is as follows: 1.77 mm sodium phosphate buffer (pH 5.8), 1.5 mm MgSO_4_, 2.0 mm Ca(NO_3_)_2_, 3.0 mm KNO_3_, 50 *μ*m Fe-EDTA, 10.3 *μ*m MnSO_4_, 1.0 *μ*m ZnSO_4_, 1.0 *μ*m CuSO_4_, 130 nm CoCl_2_, and 24 nm (NH_4_)_6_Mo_7_O_24_ (Takano et al. 2005; Yoshinari and Takano 2020). In each experiment, various concentrations of boric acid were supplied. Plants were grown at 22 to 24 °C under long-day conditions (16 h of light and 8 h of darkness).

For GUS staining, the plants were grown in vertically placed plates with solidified medium containing 1 *µ*m boric acid, 1% (w/v) sucrose, and 1.5% (w/v) gellan gum for 3 wk before being transferred to rockwool blocks (Grodan). Then, plants were grown for 9 d with B-free medium until bolting. The plants were further grown with liquid medium containing 0.3, 30, or 300 *µ*m boric acid.

For the observation of anther cross-section, plants were grown in vertically placed plates with solidified medium containing 100 *µ*m boric acid, 1% (w/v) sucrose, and 1.5% (w/v) gellan gum for 10 d and then transplanted to rockwool (Grodan) and grown with liquid medium containing 0.3 *µ*m boric acid.

For the observation of BOR1-GFP and GFP-NIP7;1 in various anther stages, plants were grown hydroponically as described previously (Takano et al. 2001). Plants were grown with liquid medium containing 30 *µ*m boric acid for 4 wk and then shifted to the medium containing 3 *µ*m boric acid and grown for 2 more wk before observation.

For the comparison of BOR1-GFP and BOR1(K590R)-GFP in different anther stages and under various B concentrations, plants were grown hydroponically with liquid medium containing indicated concentrations of boric acid for 6 to 8 wk. For phenotypic analyses, plants were grown hydroponically with liquid medium containing 100 *μ*m boric acid for 4 wk until bolting. Subsequently, they were transferred to the medium containing 20 *μ*m boric acid and grown until they were 7 wk old.

For inflorescence stem grafting, plants for rootstocks and scions were grown hydroponically with liquid medium containing 100 *μ*m boric acid until the height of the bolt reaches 5 to 10 cm. After grafting, plants were grown with medium containing 20 *μ*m boric acid until they were 2 mo old.

### Confocal microscopy

For PI/FDA staining, pollen grains were applied onto glass slides with the staining solution containing 1 *μ*m PI (Fujifilm Wako Pure Chemical) and 2.5 *μ*m FDA (Invitrogen). After incubating for 5 min, pollen grains were observed under a Leica TCS SP8 system equipped with a DMI6000B inverted microscope, 2 HyD hybrid detectors, and 2 photomultiplier tubes (PMTs), along with a 20× water immersion objective lens (numerical aperture = 0.75). The laser excitation and spectral detection bandwidths were 488 and 500 to 530 nm (laser power: 0.1% to 0.2%; gain: 20% for HyD) for FDA and 552 and 600 to 630 nm (laser power: 1.0% to 2.0%; gain: 100% to 200% for HyD) for PI.

For the observation of BOR1-GFP, BOR1(K590R)-GFP, and mRFP-ARA7 in anthers, young buds from the transgenic plants were applied onto glass slides and observed under the Leica TCS SP8 system with the 40× water immersion objective lens. The laser excitation and spectral detection bandwidths were 488 and 500 to 530 nm (laser power: 0.3% to 8.0%; gain: 100% for HyD) for GFP, 488 and 650 to 700 nm (laser power: 0.8% to 8.0%; gain: 50% for HyD or 600 V for PMT) for chlorophyll autofluorescence, and 552 and 560 to 630 nm (laser power: 2.9%; gain: 100% for HyD) for mRFP. Anther cross-section was prepared using the method outlined in a previous report (Yoshinari and Takano 2020). Briefly, fresh flower buds were embedded in 2.5% (w/v) gellan gum and cut with a layered razor blade.

For the observation of tapetal cells, young buds were applied onto glass slides with a chloral hydrate solution (8 g of chloral hydrate, 1 mL of glycerol, and 2 mL of water), and differential interference contrast images were acquired using 40× water immersion objective lens.

### Subcellular localization analysis

For the analysis of the colocalization of BOR1-GFP and mRFP-ARA7, regions of interest (ROIs) measuring 10 × 10 *μ*m were defined in cytoplasmic regions containing punctate structures. Pearson's and Spearman's correlation (PSC) coefficients were calculated between BOR1-GFP images and original or flipped images of the mRFP-ARA7, using ImageJ (https://imagej.net/Fiji) with the PSC colocalization plugin (French et al. 2008).

For the analysis of the polar localization BOR1-GFP in tapetum, ROIs measuring 5 *µ*m in length and 2 *µ*m in width were defined on the inner (locule side), lateral (between tapetal cells), and outer (middle layer-side) PM. Signal intensities (mean gray values) within the ROIs were measured for each cell. For the lateral signal intensity, the sum of the signals from both lateral sides of the cell was divided by 4, because the lateral signal includes fluorescence from the lateral PM of adjacent cell. To normalize differences in BOR1-GFP expression levels between cells, each value was divided by the sum of the intensities in the 3 regions (inner, outer, and calculated lateral).

### GUS staining

Flower tissues were incubated in 90% (v/v) ice-cold acetone for 15 min. After removing acetone, the specimens were incubated in GUS staining buffer (50 mm sodium phosphate buffer [pH 7.2], 0.5 mm K_3_(Fe[CN]_6_), 0.5 mm K_4_(Fe[CN]_6_), and 1 mm X-Gluc) overnight at 37 °C. The specimens were washed by 70% (v/v), 50% (v/v), and 20% (v/v) ethanol in this order. Then, the specimens were incubated in 10% (v/v) ethanol/50% (v/v) glycerol solution for 10 min and transferred to 50% (v/v) glycerol without ethanol.

### Inflorescence stem grafting

Inflorescence stem grafting was conducted as previously described (Nisar et al. 2012). Plants were grown hydroponically until the height of the bolt reached 5 to 10 cm. The plants for rootstocks were cut at 2 to 5 cm above the rosette, and silicone tubes (1.5 mm internal diameter, AS ONE) were cut into 1.5 cm lengths and placed over the rootstock stems. A vertical incision of 0.5 to 1 cm deep was made on each rootstock stem tip. The scions were cut from the plants, and the cut ends were trimmed into wedge shapes. The scion wedges were placed into the incisions in the rootstocks, and the silicone tubes were slid over the junction. The junctions were then covered by Parafilm (Amcor). Throughout the grafting procedure, sterile water was poured onto sections to avoid drying.

### Scanning electron microscopy

For SEM, pollen grains from plants grown for 6 to 8 wk were applied on adhesive carbon tape. The samples were coated with palladium-gold using E-1010 ion sputter (Hitachi high-tech) and observed under SU1510 SEM (Hitachi high-tech).

### LA-ICP-MS

For LA-ICP-MS analysis, pollen grains from plants grown for 6 to 8 wk were applied onto double-sided tape attached to glass slides. The slides were applied in a laser ablation system NWR 213 (Nd: YAG 213 nm; Elemental Scientific Lasers) combined with an ICP-MS (Agilent 8800 ICP-MS/MS, Agilent technologies). A laser pulse was used to individually vaporize pollen grains, and their contents were analyzed using ICP-MS. Signal intensity of ^11^B from each pollen grain was monitored at the integration time of 0.5 s and was normalized by the signal intensity obtained from a standard glass material (NIST 612; National Institute of Standards and Technology). Details of operational parameters were as follows: laser energy: 0.6 to 1.3 J/cm^2^ for pollen, 2.5 to 3.5 J/cm^2^ for NIST 612; spot size: ϕ35 *μ*m for pollen, 50 × 50 *μ*m^2^ for NIST 612; dwell time: 2 s for pollen, 10 s for NIST 612; laser repetition rate: 20 Hz; ICP incident power: 1,600 W; signal integration time: 0.5 s; plasma gas flow: 15 L/min; and He carrier gas flow: 0.8 L/min.

### Statistics

For each experiment, statistical analyses were performed using GraphPad Prism 8 software (GraphPad Software). Statistical significances were determined with the Student's *t*-test or Tukey's multiple comparisons test as indicated in the figure legends.

### Accession numbers

Sequence data from this article can be found in the Arabidopsis Information Resource GenBank/EMBL data libraries under accession numbers_*BOR1* (AT2G47160) and *NIP7;1* (AT3G06100).

## Supplementary Material

kiaf100_Supplementary_Data

## Data Availability

The data underlying this article will be shared on reasonable request to the corresponding author.

## Supplementary data

The following materials are available in the online version of this article.

**Supplementary Figure S1.** GUS staining of *BOR1-GUS* plants.

**Supplementary Figure S2.** Tapetal cells at Stage 8 expressing BOR1-GFP and BOR1(K590R)-GFP.

**Supplementary Figure S3.** Stage-dependent degradation of NIP7;1.

**Supplementary Figure S4.** Colocalization of BOR1-GFP and mRFP-ARA7.

**Supplementary Figure S5.** Tapetal cell morphology in Col-0 and the bor1-1 mutant.

**Supplementary Figure S6.** Pollen morphology under high-B conditions.

## Dive Curated Terms

The following phenotypic, genotypic, and functional terms are of significance to the work described in this paper:

BOR1 Gramene: AT2G47160
BOR1 Araport: AT2G47160

NIP5;1 Gramene: AT4G10380
NIP5;1 Araport: AT4G10380

NIP7;1 Gramene: AT3G06100
NIP7;1 Araport: AT3G06100

pollen AmiGo: PO:0025281

boric acid Gramene: EO:0007540
boric acid Araport: EO:0007540

boron CHEBI: CHEBI:27560
