# Supplementary material for: The polar-localized borate exporter BOR1 facilitates boron transport in tapetal cells to the developing pollen grains
Source: Plant Physiol. 2025 Mar 19;197(4):kiaf100. doi: 10.1093/plphys/kiaf100 (PMC11953027; doi:10.1093/plphys/kiaf100)
Supplement: kiaf100_Supplementary_Data [file kiaf100_supplementary_data.pdf]

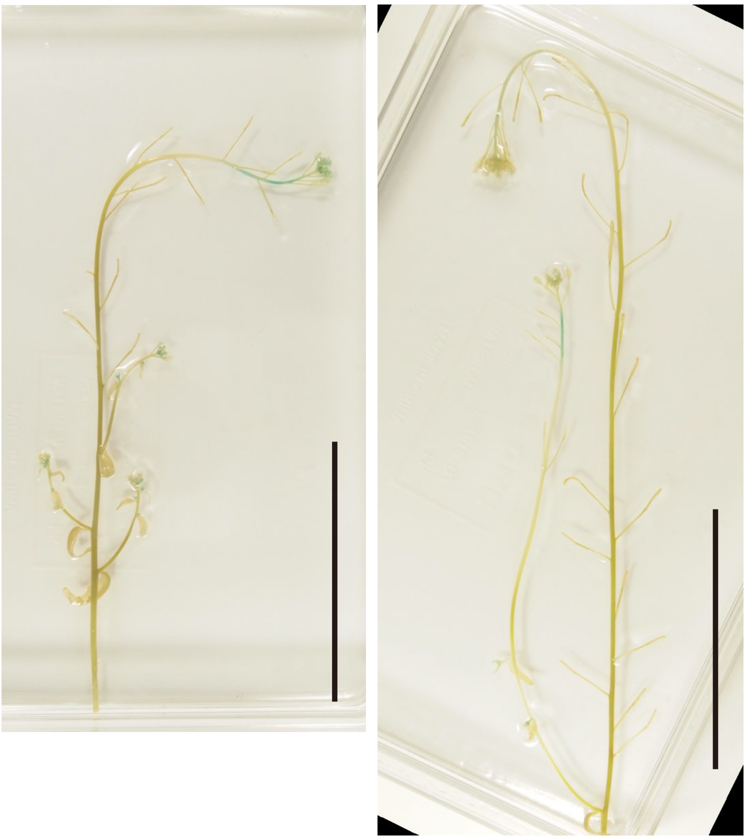

Supplementary Figure S1. GUS staining of *BOR1-GUS* plants.

GUS (β-glucuronidase) histochemical staining of the shoots of transgenic Arabidopsis *bor1-1* plants expressing *BOR1-GUS* under the control of the BOR1 promoter. Plants were grown hydroponically with the medium containing 3 μM B for 6 weeks. Bars represent 5 cm.

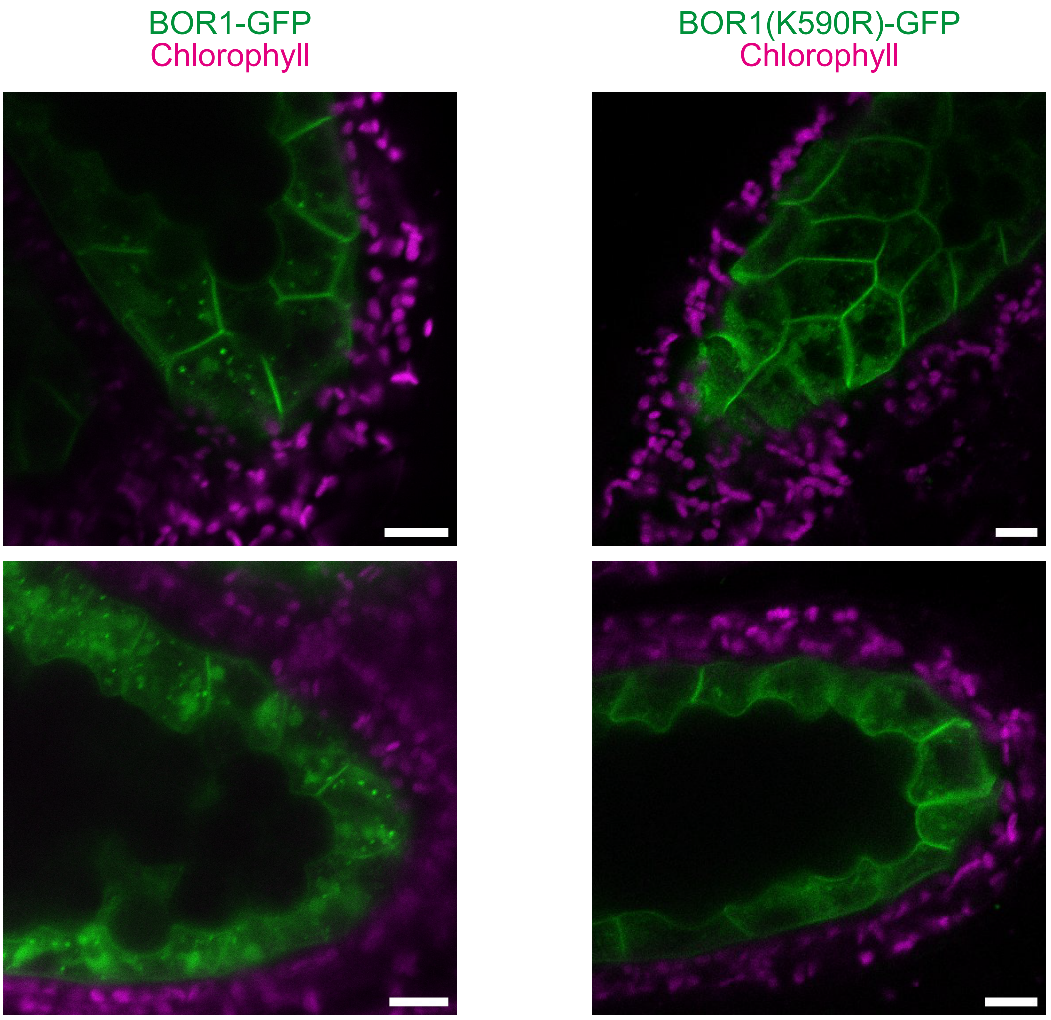

Supplementary Figure S2. Tapetal cells at stage 8 expressing BOR1-GFP and BOR1(K590R)-GFP.

Confocal images of BOR1-GFP or BOR1(K590R)-GFP (green) and chlorophyll fluorescence (magenta) in anthers at stage 8. Plants were grown hydroponically with the medium containing 3 μM B for 5 weeks. Bars represent 10 μm.

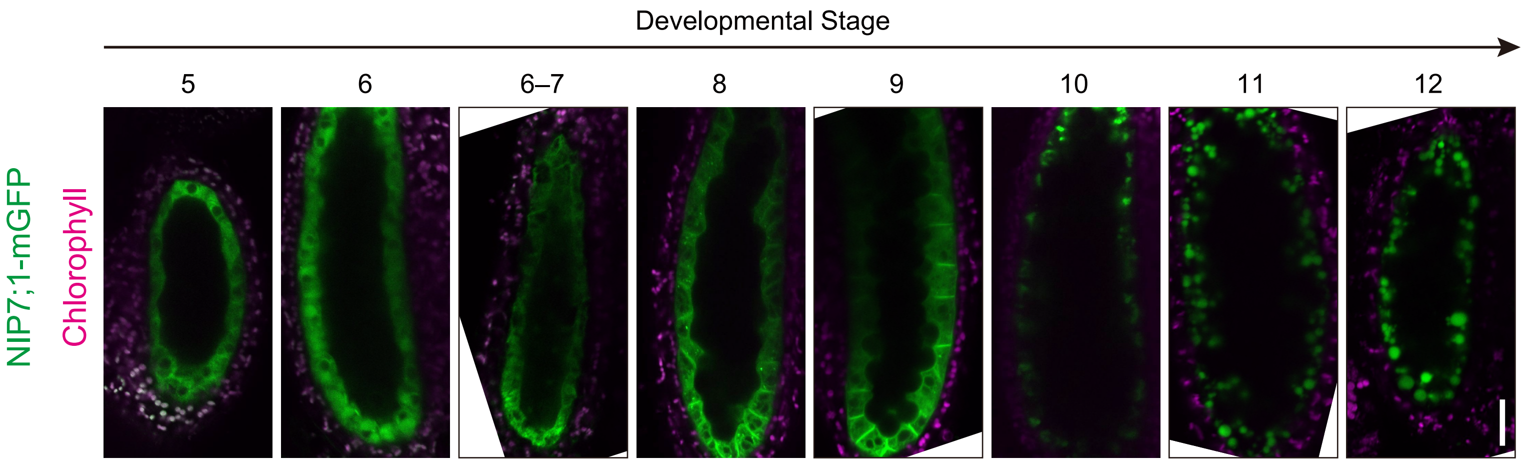

Supplementary Figure S3. Stage-dependent degradation of NIP7;1.

Confocal images of NIP7;1-mGFP (green) and chlorophyll fluorescence (magenta) in anthers at various developmental stages. Scale bar = 10 μm. Plants were grown with liquid medium containing 30 μM boric acid for 4 weeks and then shifted to the medium containing 3 μM boric acid.

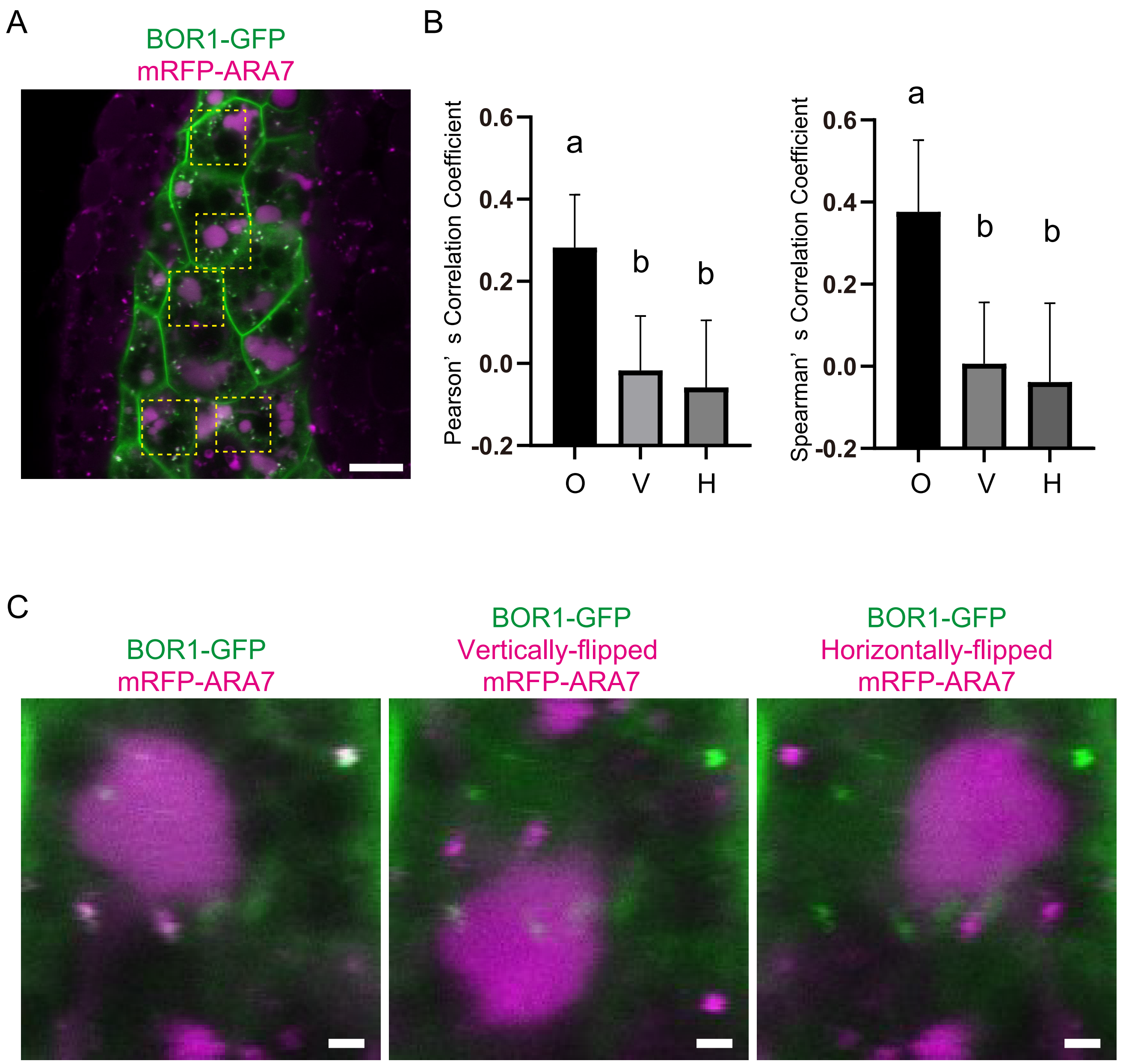

Supplementary Figure S4. Co-localization of BOR1-GFP and mRFP-ARA7.

A, A confocal image of tapetum in stage 8 expressing BOR1-GFP and mRFP-ARA7. Uncropped version of the image shown in Figure 2D. The ROIs analyzed in B are marked by dotted squares. Scale bar represents 10 μm.

B, Analysis of co-localization of BOR1-GFP and mRFP-ARA7. Pearson' s and Spearman' s correlation coefficients were calculated between BOR1-GFP and original or flipped mRFP-ARA7 images. O, original; V, vertically flipped; H, horizontally flipped. n = 5 ROIs (10 μm × 10 μm). Different letters indicate significant differences (P<0.001) by Tukey's multiple comparisons test.

C, Merged images of BOR1-GFP and original or flipped mRFP-ARA7. Scale bars represent 1 μm.

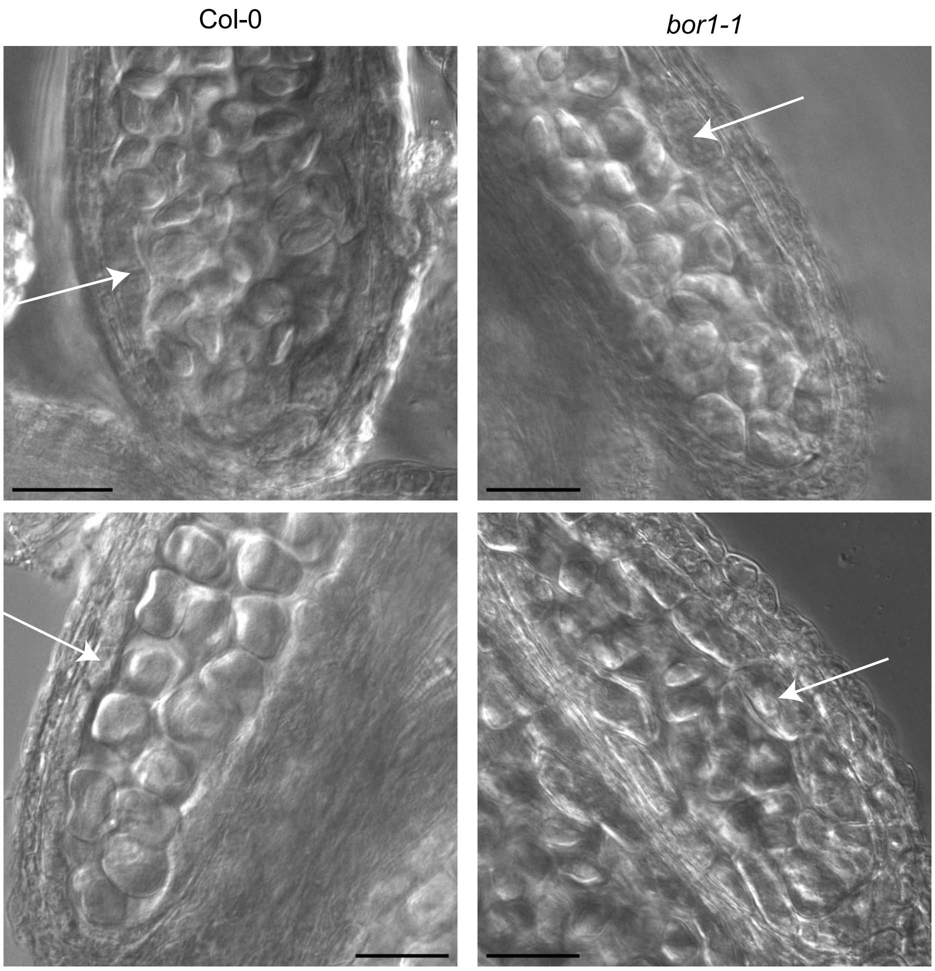

Supplementary Figure S5. Tapetal cell morphology in Col-0 and the *bor1-1* mutant. Differential interference contrast images of stage 5–7 anthers from Col-0 and *bor1-1* plants. Tapetal cells are indicated by white arrows. Bars represent 20 μm.

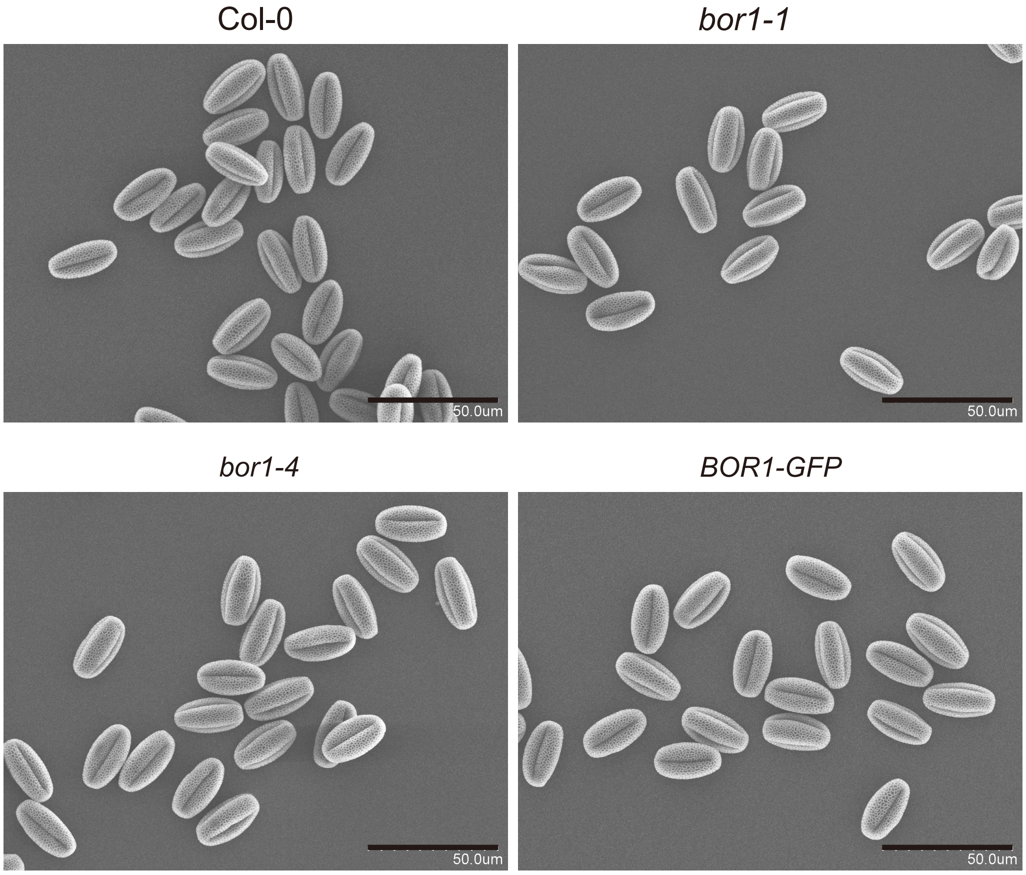

Supplementary Figure S6. Pollen morphology under high-B conditions.

Representative scanning electron microscope image of the pollen grains from Col-0, *bor1-1*, *bor1-4*, and transgenic *bor1-1* plants expressing *BOR1-GFP*. Bars represent 50 μm. Plants were grown hydroponically with the medium containing 100 μM B for 7 weeks.
